# Plant secretome proteomics

**DOI:** 10.3389/fpls.2013.00009

**Published:** 2013-02-01

**Authors:** Erik Alexandersson, Ashfaq Ali, Svante Resjö, Erik Andreasson

**Affiliations:** Department of Plant Protection Biology, Swedish University of Agricultural SciencesAlnarp, Sweden

**Keywords:** apoplast, mass spectrometry, plant, proteomics, secretome

## Abstract

The plant secretome refers to the set of proteins secreted out of the plant cell into the surrounding extracellular space commonly referred to as the apoplast. Secreted proteins maintain cell structure and acts in signaling and are crucial for stress responses where they can interact with pathogen effectors and control the extracellular environment. Typically, secreted proteins contain an N-terminal signal peptide and are directed through the endoplasmic reticulum/Golgi pathway. However, in plants many proteins found in the secretome lack such a signature and might follow alternative ways of secretion. This review covers techniques to isolate plant secretomes and how to identify and quantify their constituent proteins. Furthermore, bioinformatical tools to predict secretion signals and define the putative secretome are presented. Findings from proteomic studies and important protein families of plant secretomes, such as proteases and hydrolases, are highlighted.

## INTRODUCTION

In plant cells, many proteins undergo secretion or exocytosis to the extracellular space (ECS) in order to maintain cell structure, regulate the external environment and as a part of signaling and defense mechanisms. The ECS is composed by the cell wall and space between these and contains what is often referred to as the apoplastic fluid (APF). The protein composition of the phloem and xylem are not considered in this review.

The word secretome was first used in association with proteins secreted from bacteria (Tjalsma et al., 2000). In plants, there has been an ongoing discussion on how to define secretomics. Agrawal et al. (2010) described it as “*the global study of secreted proteins into the ECS by a cell, tissue, organ or organism at any given time and conditions through known and unknown secretory mechanisms involving constitutive and regulated secretory organelles*.” This is a useful definition even though it should be noted that until now few studies have identified more than 100 proteins and thus cannot be considered to give a “global” view of the secretome.

In “classical” or “conventional” protein secretion, a mechanism highly conserved in eukaryotes, proteins containing a signal peptide are transported via the Golgi apparatus. In the *Arabidopsis* genome, 18% of proteins are predicted to be secreted (the Arabidopsis Genome Initiative, 2000), but recurrently between 40 and 70% of the proteins identified in secretome studies lack a signal peptide, and thus putatively belong to the class of leaderless secreted proteins (LSPs), even though possible contamination of proteins from other cell compartments is a concern (discussed below).

In spite of the fact that commonly the majority of identified secretome proteins lack signal peptides, unconventional protein secretion (UPS) has been little studied in plants, as pointed out in a recent review by Ding et al. (2012). UPS can be divided into two major classes: proteins are either transported in a non-vesicular mode where they pass directly from the cytosol through the plasma membrane or by various vesicular modes with membrane-bounded structures fusing with the plasma membrane before release in the ECS. Recently, a plant-specific compartment named EXPO (exocyst-positive organelle), which appears to mediate UPS without proteins passing the Golgi apparatus, trans-Golgi network, or multi-vascular body, was discovered (Wang et al., 2010).

## ISOLATION AND IDENTIFICATION OF SECRETOME PROTEINS

Until the last 3–4 years suspension-cultured cells (SSCs) were the preferred choice for preparation of secretome samples and so far around half of the published studies have been conducted with SSC. Advantages over material derived *in planta* are that cell leakage can be readily estimated by determining the number of dead cells and that the separation of the secretome from intact cells by filtration and/or centrifugation is easier. Still, the general trend is that recent studies are carried out* in planta*, and there are good reasons for this switch since SSC does not provide a natural environment for the cells and physiologically relevant treatments are difficult to apply. Furthermore, it is possible to derive organ- and developmental-specific secretomes using plant material. Jung et al. (2008) reported on a striking difference in rice secretome proteins depending on whether *in planta* or SSC material were used with an overlap of only 6 spots out of 222 after resolution by two-dimensional gel electrophoresis (2D-PAGE) and a difference in the levels of identified proteins with predicted signal peptides of 27 and 76%, respectively, between the two systems. Both secretomes showed low level of contamination as determined by a malate dehydrogenase activity. This indicates that there are large differences in the protein populations derived *in planta* and from SSC and that the secretion mechanisms might be fundamentally different.

**FIGURE 1:**
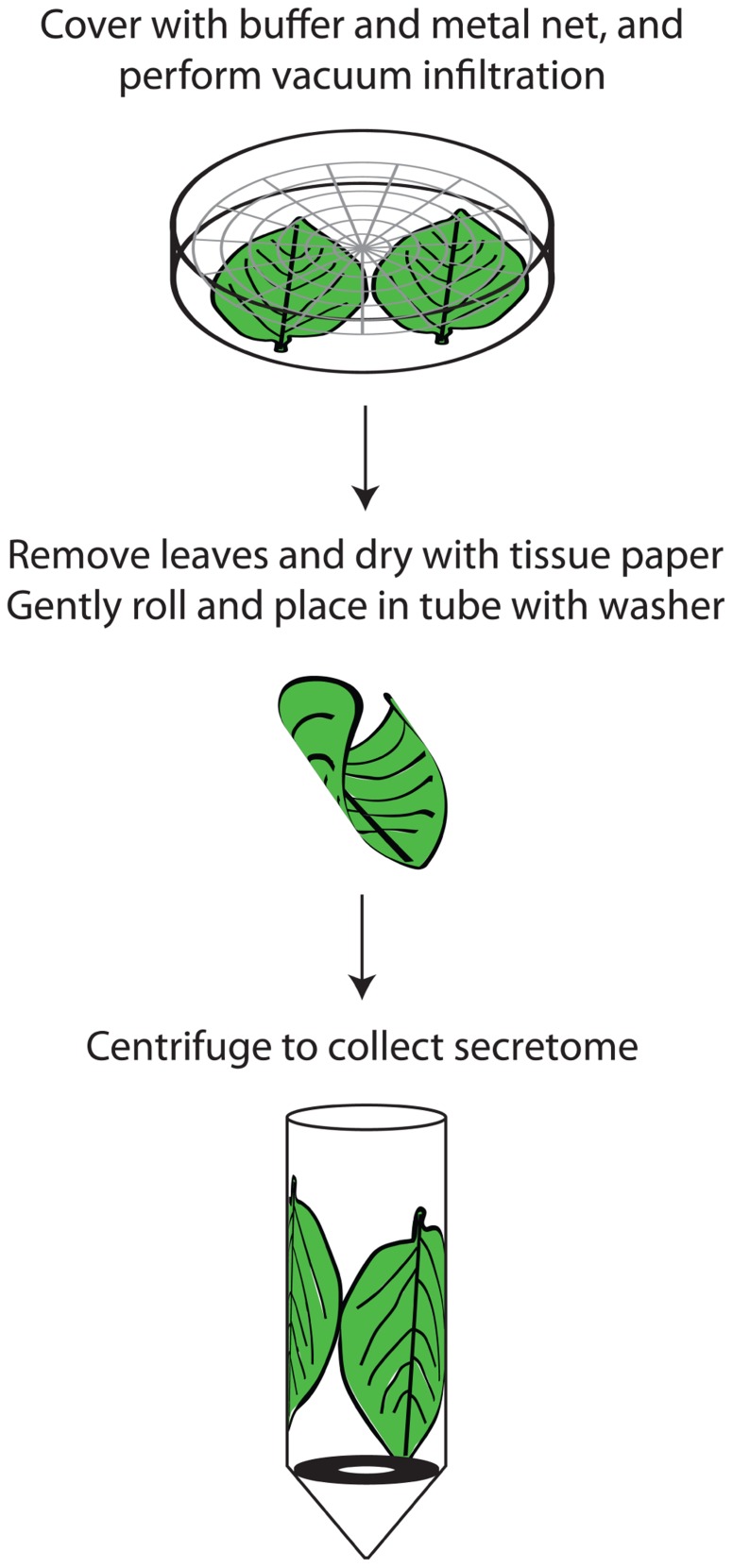
**Outline of secretome preparation in potato leaves**. The leaves are washed in a beaker by gentle swirling in a solution of 1% Tween-20 and thereafter dried quickly. Up to five potato leaves can be processed at the same time. They are then placed in petri dishes containing 150 mM sodium phosphate pH 7 and 50 mM sodium chloride and covered with a metal grid to ensure that they are completely immersed. Vacuum is applied for two rounds of 5 min each, with the pressure being allowed to return to atmospheric in between. The leaves are then removed, gently dried with tissue paper, placed in tubes containing protease inhibitor and centrifuged for 1000*g* at 4°C. It is vital to roll the leaves gently, preferably several together, when placing them in the tubes to minimize cell breakage and subsequent contamination. A metal washer in the tube separates the extracted secretome from the leaves.

When preparing secretomes from intact plant organs caution must be taken to minimize cell breakage and leakage. Non-destructive methods are less likely to give rise to cytosolic contamination. The most common method, vacuum infiltration-centrifugation, has been practiced for about 50 years (Klement, 1965). Whereas pH of the infiltration buffer affects the metabolic composition, osmolarity and incubation time have little effect on the eluate (Lohaus et al., 2001). Due to differences in sample infiltrability adjustment depending on species may be necessary, e.g., Nouchi et al. (2012) recently suggested a rice-specific method. For potato leaves we first thoroughly rinse the leaves with a buffer to reduce leaf surface tension and facilitate the vacuum infiltration which is repeated once. After infiltration the leaf surfaces should quickly be dried not to dilute samples. Thereafter carefully rolled leaves are transferred to 15 mL Falcon tubes with a washer at the bottom to avoid immersion of leaves into the collected APF (Ali et al., 2012; **Figure 1**). The centrifugation force should not exceed 1000*g* in order to avoid cell breakage (Terry and Bonner, 1980). In general, there is a noticeable lack of studies comparing the effect of different procedures, e.g., buffer concentrations, for secretome isolation.

An alternative, the gravity extraction method (GEM), has been proposed, but it has so far only been used in one study (Jung et al., 2008). In this method, the vacuum infiltration step is omitted and the APF is collected directly to decrease cell damage and solubilization of membrane proteins. After sap isolation it is necessary to add a cetyltrimethylammonium bromide (CTAB) precipitation step to remove interfering compounds such as carbohydrates. Various methodological adaptations might be necessary for efficient secretome preparation, e.g., a water-displacement method was developed to obtain apoplast fluid from stem tissue in poplar (Pechanova et al., 2010).

## PURITY ASSESSMENT

Enzyme activity, immunoblotting and microscopy have been used to assess the purity of secretome fractions, e.g., by comparison to the microsomal fraction. Especially *in planta* studies require more stringent assessment of purity to ensure secretome fractions with little intracellular contamination. To estimate cytosolic contamination, enzyme activities of glucose-6-phosphate dehydrogenase, catalase and malate dehydrogenase are commonly measured. Based on malate dehydrogenase activity 1–3% of contamination is usually seen, but up to 10% has been reported (Song et al., 2011). In destructive methods, where the cell-wall fraction is isolated, determining the putative contamination by the plasma membrane is also necessary, e.g., by measuring H-ATPase activity (Pandey et al., 2010; Bhushan et al., 2011). Antibodies against malate dehydrogenase and RuBisCo are also frequently used to determine the contamination level (Pechanova et al., 2010; Gupta et al., 2011). It can be necessary to estimate levels of membrane damage caused by the test condition itself, and for this purpose electrolyte leakage and concentration of malondialdehyde, which is a breakdown product of membrane lipid peroxidation, have been measured (Zhou et al., 2011). In plant–pathogen interaction studies, it should be remembered that cell leakage can be caused by direct damage of hyphae or cell wall maceration, and thus be a result of the biological system itself rather than the isolation procedure. Likewise, plant developmental processes, such as programmed cell death during xylem formation, can release non-secretary cytosolic proteins into the apoplast.

By estimating enrichment or depletion of peptides of a set of marker proteins, quantitative proteomics can be a good method to determine the level of contamination. Due to its sensitivity, selected reaction monitoring mass spectrometry (SRM-MS), discussed below, is particularly promising.

## PROTEOMIC ANALYSES

In plant secretome analysis mainly 2D-polyacrylamide gel electrophoresis (PAGE), but also 1D sodium dodecyl sulfate (SDS)-PAGE, have been used (e.g., Gupta et al., 2011; Ali et al., 2012). 2D-PAGE is a well-established and relatively inexpensive method. While transmembrane, highly hydrophobic and very large proteins can be difficult to analyze using 2D-PAGE, apoplast proteins do not generally belong to these categories. Furthermore, 2D-PAGE separation is based on the properties of the intact proteins and splicing and post-translational modifications (PTMs) will affect migration, something that is not always desirable. Finally, multiple proteins are often identified from the same 2D-gel spot, making unambiguous identification difficult.

More recently, high-performance liquid chromatography (HPLC)-based methods, where tryptic peptide digests rather than proteins are analyzed, permit simultaneous identification of larger number of proteins. The proteins can be digested directly or pre-fractionated, e.g., on 1D-SDS-PAGE. Consequently, HPLC-based methods are useful for the analysis of hydrophobic proteins difficult to retain in solution during 2D-PAGE and for small proteins such as the proteolytic fragments commonly found in the protease-rich apoplast. However, these methods sometimes require more complicated sample preparation and data processing, and since analysis is done for individual peptides splice variants and PTMs may remain unrecognized.

In the HPLC-based methods, peptides can be quantified either by isotopic labeling or by label-free methods (Schulze and Usadel, 2010; Neilson et al., 2011; Yao, 2011). Isobaric tags for relative and absolute quantitation (iTRAQ) was used by Kaffarnik et al. (2009) to analyze secretome of *Arabidopsis* SSC challenged with *Pseudomonas*. In the label-free methods, quantitative data is obtained by analyzing the intensity of the mass spectrometrical signal from a peptide, or by counting the number of times it is identified (spectral counting). The label-free methods are simpler in terms of sample preparation and the number of comparisons you can make is not limited, but analysis can be more computationally challenging.

In SRM-MS, the eluate from an HPLC column is monitored to detect selected peptides enabling high dynamic range (Anderson and Hunter, 2006; Kitteringham et al., 2009). When combined with isotopically labeled internal standard peptides this method allows for sensitive absolute quantification. However, it will only measure peptides from a pre-selected set.

In plant–pathogen interactions identification of secreted proteins from more than one organism is expected. Still, very few pathogen proteins have been identified in interaction studies. Nevertheless, since proteins from the interacting organism can be expected to be a minority, precautions should be taken to avoid false positive hits from host peptides when matching pathogen peptides. In our experience, the use of a combined plant–pathogen protein database extended with a random sequence database for false discovery rate determination is a good approach.

Pathogens and other sources of stress result in a powerful oxidative burst in the secretome. Protein oxidation is known to affect both stability and enzymatical activity (Sweetlove and Moller, 2009) and oxidation proteomics is a field in rapid development. Oxidation products can be identified in global analyses by searching for peptides with oxidized amino acids, or by enrichment-based approaches, e.g., enrichment of carbonylated (Madian and Regnier, 2010) or nitrosylated peptides (Lindermayr et al., 2005). Since oxidative modifications can be quite labile, sample preparation should be optimized to minimize changes in oxidation state (Hawkins et al., 2009). Depending on the possible enrichment strategies, buffer composition should be considered during experimental design, e.g., when isolating modified cysteine residues (Lindermayr et al., 2005). A proteomic investigation of oxidation in the secretome is still lacking but could yield interesting knowledge regarding targets and extent of the oxidative burst.

## BIOINFORMATICAL TOOLS AND DATABASES

For *in vitro* prediction of signal peptides SignalP (Petersen et al., 2011) has been widely used. SecretomeP is a prediction method trained on sequence features outside of the signaling peptide of secreted proteins (Bendtsen et al., 2004). It is based on mammalian and bacterial proteins, but interestingly, 60% of the LSPs identified in *Arabidopsis* SSC were predicted to be secreted by SecretomeP (Cheng et al., 2009). New tools are emerging and, e.g., LocTree2 has high prediction success especially for secreted proteins (Goldberg et al., 2012).

In the SUBA3 database 471 *Arabidopsis* proteins are registered as “extracellular” based on MS/MS identification (Heazlewood et al., 2007). Little less than half of these have been reported exclusively in the “extracellular” compartment and less than 10% are not predicted by TargetP to be extracellular proteins. Lum and Min (2011b) identified 1704 plant proteins annotated as secreted in the manually curated UniProt database. Using three prediction tools 97.5% were identified to carry a signal peptide. A database for secreted plant proteins, PlantSecKB, is currently being established (Lum and Min, 2011a).

## BIOLOGICAL FINDINGS

For plant secretome studies published before 2010 we mainly refer to Agrawal et al.’s comprehensive review (Agrawal et al., 2010). Since then more than a dozen studies have appeared (highlighted below). Protein families commonly found in the secretome are listed in **Table 1**. For a review on secretomes of oomycetes and fungi we refer to Kamoun (2009).

**Table 1:** Proteins families commonly found in the secretome. Protein family name is given together with PLAZA2.5 gene family identifiers and number of members in *Arabidopsis* and rice (spp. *japonica*) according to PLAZA2.5.

Protein family	PLAZA2.5 gene family identifier	Number identified in *A. thaliana*	Number in rice (spp. *japonica*)
Subtilase-related (e.g., P69 protein, proteinase inhibitor I9, serine proteases)	HOM000020	69	77
Eukaryotic aspartyl protease	HOM000037	58	102
Serine carboxypeptidase	HOM000054	54	60
Cysteine protease inhibitor (serpins)	HOM000290	13	20
Pectin methylesterase inhibitor/ Pectin lyase-like	HOM000034	70	47
Kunitz-type trypsin and protease inhibitor	HOM000489	4	3
Glycosyl hydrolases family 17 (e.g., PR2)	HOM000021	95	86
Glycosyl hydrolases family 31	HOM000508	5	6
Chitinase family (Glycoside hydrolase, family 19, PR3)	HOM000272	14	18
Cysteine-rich secretory protein family (e.g., PR1)	HOM000169	23	40
Thaumatin family (e.g., PR5)	HOM000107	22	36
Pectinacetylesterase	HOM000436	12	10
RmlC- and germin-like cupins	HOM000084	33	42
Peroxidases class III	HOM000453	7	10
xyloglucan endotransglucosylase/hydrolase	HOM000089	33	30

Plant secretomes have been studied in natural conditions (e.g., Soares et al., 2007), in different cultivars (e.g., Konozy et al., 2012), during nutritional deficiency (Tran and Plaxton, 2008), after hormone treatment (e.g., Cheng et al., 2009), temperature change (Gupta and Deswal, 2012), salt stress (Song et al., 2011), and presence of pathogens and elicitors (e.g., Kim et al., 2009).

Martinez-Esteso et al. (2009) studied the grape secretome of SSC in response to methylated cyclodextrins and methyl jasmonate (MeJA) and could show that the expression levels of peroxidases, pathogenesis-related (PR) proteins, SGNH plant lipase-like, xyloglucan endotransglycosylase and subtilisin-like protease were affected. In a similar study, application of elicitors MeJA and cyclodextrins also led to the identification of chitinases and other PR proteins in tomato SSC (Briceno et al., 2012).

Gupta et al. (2011) characterized the secretome from SSC of the legume chickpea and identified over 700 proteins by combining 1D SDS-PAGE and HPLC-MS/MS. By comparing the secretome based on sequence homology to previously published *Arabidopsis*, *Medicago*, and rice data the authors could show a large degree of species-specificity in secreted proteins hinting at differences in the apoplast composition between species and monocots and dicots, something that needs further investigation. Cultivar-specific secretome composition also exists and in the fruit pericarp of three tomato cultivars the percentage of proteins with signal peptides varied with 50–70% (Konozy et al., 2012).

Even if only a few proteins were identified, differences in the effects on exocytosis and protein transport were observed in an elegant experiment using transient over-expression of different SNAREs in tobacco protoplasts (Ul-Rehman et al., 2011)

Several studies have targeted the rhizosphere. Over 100 secreted proteins were identified from rice roots grown in an aseptic hydroculture (Shinano et al., 2011). These proteins are believed to play an important role in the rhizosphere and a relatively high number (54%) had predicted signal peptides. Ma et al. (2010) collected proteins secreted in the mucilage of primary maize roots. Using a combination of 1D SDS-PAGE and HPLC-MS/MS, the presence of 2848 proteins were reported, which is over 50 times more compared to earlier quantitative studies of root mucilage based on 2D-PAGE or MudPIT (Basu et al., 2006; Wen et al., 2007).

The effects in the secretome of rice seedlings were studied under oxidative stress caused by 0.3 and 0.6 mM of hydrogen peroxide (H_2_O_2_). Of the 54 proteins identified, around half of the responsive proteins were involved in carbohydrate metabolism, with redox homeostasis as the second largest group (Zhou et al., 2011). The typical stress response marker PR1a was also up-regulated. In rice leaves more than 100 identified proteins were shown to be affected by drought stress in a time series spanning over 8 days (Pandey et al., 2010). Similarly to this study, Song et al. (2011) studied the effect of salt stress in rice during a 12 h time course and found 64 proteins with changed abundance. In both studies, proteins related to carbohydrate metabolism were the largest group of proteins with changed abundance.

Gupta and Deswal (2012) explored the secretome of seabuckthorn after low-temperature treatment and identified thaumatin-like protein and chitinase as putative antifreeze proteins. Pechanova et al. (2010) collected secretome samples and measured gene expression by microarrays from poplar growing in a riverine ecosystem exposed to multiple stresses. The composition of the secretome showed clear specificity depending on the tissue and type of stress response.

In plant interaction studies, Goulet et al. (2010) found around 90 proteins, two of which were bacterial, in the leaf apoplast of *Nicotiana benthamiana* infected by the bacterial gene vector *Agrobacterium tumefaciens*. PR proteins were found to be the most abundant proteins in the isolated fraction, and several increased greatly upon infection. Floerl et al. (2012) identified seven proteins, several of which were peroxidases, with changed abundance in the leaf secretome of *Arabidopsis* infected by the soil-borne fungal pathogen *Verticillium longisporum*. Shenton et al. (2012) used virulent and avirulent *Magnaporthe oryzae* strains to compare compatible and incompatible interactions in rice in early and late infection. A number of DUF26 domain-containing proteins increased in the compatible interaction already at 12 h. In the incompatible reaction several PR proteins were accumulated. Interestingly, one *M. oryzae* protein, a cyclophilin, was identified and that only in the compatible interaction. The authors reduced the detergent concentration in the vacuum infiltration buffer to compensate for Tween-20 used for the *Magnaporthe* inoculation.

## CONCLUSIONS AND FUTURE PERSPECTIVES

To date, over 30 secretome studies in more than 10 plant species have shown that hundreds of proteins are secreted into the apoplast. The relatively simple procedure to isolate secretome samples together with the fact that it constitutes the interface between the plant cell and its environment makes it an excellent fraction for identification of biomarkers for signal and stress cues, and highly suitable for monitoring biotic interactions. Secretome studies have firmly established the presence of a substantial level of secreted proteins lacking signal peptides and indicated a large degree of plant species specificity in the composition of secreted proteins. A transition from SSC to *in planta* systems have taken place, but comparative organ-specific studies are still lacking and little is known about the changes in the secretome during plant developmental stages, which are known to affect both metabolism, signaling pathways and resistance levels. Finally, no global study has been done of glycosylation of secreted proteins and little is known of PTMs, such as oxidation, in this fraction. To identify putative effector targets in the secretome, reliable quantitative proteomics will be crucial, since a down-regulation of a protein upon pathogen attack might indicate regulation by pathogen effectors.

Recent technical advances such as improved databases, e.g., based on RNA-seq data, and increased sensitivity of mass spectrometers will aid in the identification of specific isoforms. The regulation of single gene family members important in ecological and agricultural systems can now be dissected even in non-model species. Furthermore, the high through-put of SRM-MS will enable processing of large sample numbers, e.g., by so called ecoproteomics in field-grown material exposed to complex, natural environments, and influenced by multiple organisms. Overall, we are closer than ever to global analyses of plant secretomes similar to what we have seen for some prokaryotes.

## Conflict of Interest Statement

The authors declare that the research was conducted in the absence of any commercial or financial relationships that could be construed as a potential conflict of interest.
